# Challenges and opportunities of bovine milk analysis by mass spectrometry

**DOI:** 10.1186/s12014-016-9110-4

**Published:** 2016-04-19

**Authors:** Aparna Verma, Kiran Ambatipudi

**Affiliations:** Department of Biotechnology, Indian Institute of Technology Roorkee, Roorkee, Uttarakhand 247667 India

**Keywords:** Bovine, Milk, Proteomics, Lipidomics, Mass spectrometry, Livestock

## Abstract

Bovine milk and its products (e.g. cheese, yoghurt) are an important part of human diet with beneficial effects for all ages. Although analyses of different milk components (e.g. proteins, lipids) pose huge challenges, the use of mass spectrometric (MS)-based techniques is steadily improving our understanding of the complexity of the biological traits that effect milk yield and its components to meet the global demand arising from population growth. In addition, different milk constituents have various applications in veterinary research and medicine, including early disease diagnosis. The aim of the review is to present an overview of the progress made in MS-based analysis of milk, and suggest a multi-pronged MS strategy to better explore different milk components for translational and clinical utilities.

## Introduction

Agriculture, a pivotal sector for ensuring food and nutritional security, is undergoing a radical change in India and at the global level. Conventional crop and animal production methods are facing enormous pressure related to increased grain and animal production to meet the growing demand of population increase [1]. Although Indian agriculture performed better than expected during the global food crisis in 2008, the agriculture sector needs to envision future challenges as potential opportunities to make it more sustainable to provide food security and alleviate poverty [2].

In India, livestock as a sub-sector of agriculture contributes significantly to the economy by ranking first in world milk production, as well as producing vast amounts of milk products, meat, eggs, wool, hide and skin [3]. Livestock in spite of sustained pressure from climate change and increased demand of animal protein has consistently contributed significantly to the agricultural gross domestic product. For example, animal husbandry involves approximately 5.5 % of the total work force in the country, as well as providing gender equity and women empowerment [4].

There is no program in place anywhere in the world including India that considers appropriate husbandry practices to develop milk as functional food by altering its individual components which has been previously reported to have significant association with genotype [5] and environmental factors [6]. Therefore, to keep up with the demand and supply chain of animal products, it is critical to understand the challenges for improving animal health, production, and their welfare by adopting better husbandry and management practices [7]. In particular, early and quick disease diagnosis, especially at farms is a huge challenge for veterinary physicians.

The advancement of proteomics technology has enabled researchers to analyze different body fluid such as milk [8] saliva [9] and urine to better understand etiology and pathogenesis of disease. Although the use of mass spectrometry (MS)-based proteomics in translational veterinary research is steadily increasing, information about the frequency, onset and progression of different markers (e.g. proteins, lipids) due to exogenous (e.g. season) and endogenous (age, lactation) factors which influence the dynamic nature of different milk components have not been sufficiently explored. Thus, it is critical to consider these normal differences in expression when searching for clinically relevant, disease specific markers. In this review, we provide an update on the progress made in the application of MS-based proteomics over the last 5 years in bovine milk analysis, as well as point out the possible challenges and considerations for improving livestock production and management.

## Alternate diagnostic body fluid

Historically, blood has been used as the first choice of body fluid for analyzing changes in its constituents associated with pathophysiological conditions. However, due to the limitations of analyzing low abundance proteins in blood due to its complex nature, it is imperative to explore alternate diagnostic fluids such as milk, urine, and saliva to reflect local or systemic illness. In addition, due to the variability of the sources and composition of body fluids, different approaches are required to compile a comprehensive catalogue of potential markers. To this end, MS-based proteomic methods have great potential because they are unbiased and require no prior knowledge of fluid composition. In the context of this review, milk as an alternate diagnostic fluid including its different components has been discussed with respect to its diverse applicability in livestock proteomics.

### Milk

**Fig. 1:**
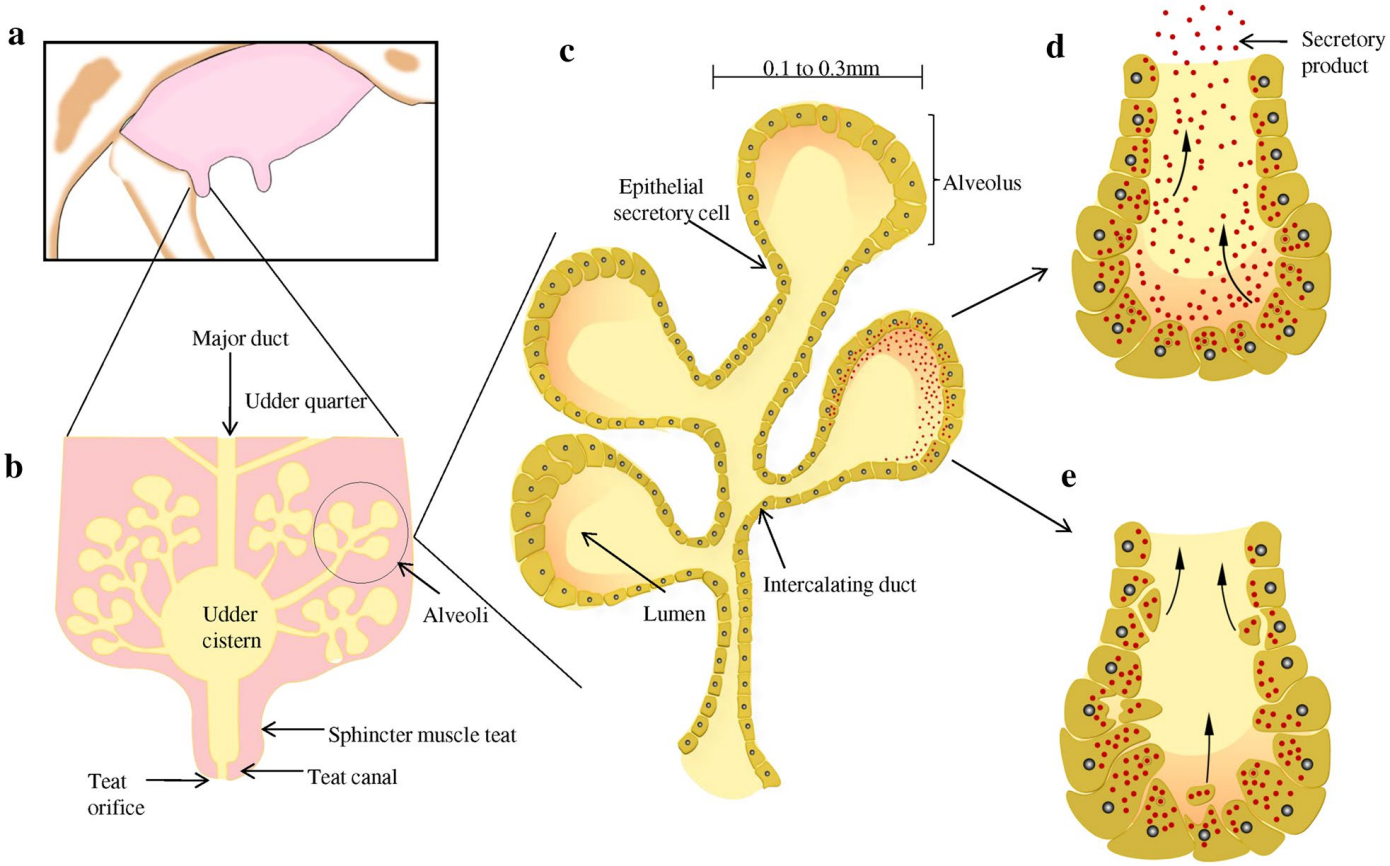
Schematic representation of the structure of mammary gland. **a** A general model of udder, **b** image of udder with complex tissue comprised of many ducts and alveoli, **c** an alveolus comprising of many cell types such as secretory and intercalating ducts, **d** an alveolus showing merocrine mode of secretion for protein component of the milk, **e** an alveolus showing apocrine mode of secretion for lipid component of the milk

Bovine milk is a complex biological fluid secreted by a dynamic and complex organ composed of various cell types working together for synthesis and secretion of milk as shown in Fig. 1. Milk is responsible with multifaceted functionality for the nourishment of young and provides a vital source of nutrition for humans of all ages. Bovine milk composition is dynamic in nature containing proteins and peptides, lipids, and complex carbohydrates with health benefits beyond the expected nutritional content. Its composition varies continuously due to different factors such as breed, feed, age, season and stage of lactation [11–13]. Although milk has evolved as a natural food under selective pressure to meet nutritional needs of different species, limited knowledge is available about changes in its components (e.g. proteins, lipids) in health and disease due to different environmental and physiological factors. Changes in the expression of these components alter normal functional properties of milk and would be expected to be indicative of systemic or local illness. However, much of the studies to date, have focused on the alteration of different milk components of exotic breeds (e.g. Jersey and Holstein–Friesian) [14, 15] with limited reports on Indian pure breed cows (e.g. Sahiwal, Tharparker) and buffaloes (e.g. Murrah, Jafarabadi), which are large contributors to Indian dairy industry.

### Protein markers

Over the past decade, a number of groups using proteomics methodologies have made significant progress in characterizing abundant milk whey proteins [16–18], while detection of medium to low abundant proteins has been a bottle-neck due to its dynamic nature [19–21]. Similarly, early detection of mastitis, inflammation of the mammary gland by biomarkers or patterns of biomarkers has had limited success [7]. Mastitis, both clinical and subclinical, is the most devastating bovine disease causing staggering economic losses worldwide to the dairy industry. Unhygienic milking practices, diverse production systems, inadequate treatments and other factors are contributing to higher incidence of mastitis [22], while the lack of early diagnostic test has led to a lag where symptoms precede diagnosis by weeks and months resulting in spread of infection to other uninfected udder and cows [18, 23]. Currently, diagnosis of mastitis relies on visual signs such as redness, swelling of the infected quarter or altered consistency of milk (thickened or watery), increased somatic cell count (SCC) or clots [24]. In contrast, the subclinical form of mastitis is more difficult to diagnose due to lack of visual signs either in the udder or in milk and is generally based on the detection of bacteria or SCC in milk [25] measuring electrical conductivity [26], lactate dehydrogenase activity [27, 28], and decreased milk production [25].

Although the majority of previous studies have reported analysis of milk and its different components, in-depth analysis requires newer technologies such as proteomics. To date, the majority of bovine milk protein analysis including post-translational modifications of different proteins (PTMs) from healthy and diseased animals have been performed using 2-dimensional gel electrophoresis (2DE), differential gel electrophoresis (2DE-DIGE) followed by MALDI-time-of-flight (TOF)-MS, and/or liquid chromatography coupled tandem mass spectrometry (LC–MS/MS) [16–18, 29–32]. In addition, to maximize protein identifications and expand the analysis of the milk proteome, multiple analytical approaches including fractionation techniques have been adopted. For example, casein which makes up 80 % of overall milk protein content was extracted using hydrophobic and hydrophilic procedures followed by size exclusion fractionation to identify low molecular weight molecules [33]. Similarly, enhanced identification of whey proteins was reported after precipitation of casein [34, 35]. Nissen et al. [19] performed different fractionation techniques such as acidification, filtration, and centrifugation followed by LC–MS/MS and identified 635 bovine whey proteins. Similarly, Molle et al. [36] applied electrospray (ESI) and matrix-assisted laser desorption (MALDI) ionization in parallel for complementary proteome coverage in bovines. Using LC–MS/MS Boehmer et al. [37] were successful in identifying proteins from complex mixtures, while Zhang et al. [17] reported change in abundance of acute phase protein abundance in colostrum and mature milk. Similar to shotgun proteomics, MALDI-TOF has gained success in bovine milk proteomics, for example, molecular weight of proteins was determined without any fractionation [38] MALDI-TOF has also been used to determine N-linked glycosylation patterns for milk proteins of milk-fat-globules [39], immunoglobulins [40], α-lactalbumin [41, 42], κ-casein [43] and lactoferrin [44, 45]. Furthermore, MALDI-TOF has achieved success in determining changes in N-glycans in early lactation [40] as well as top-down sequencing of complex O-glycans at the protein level [46].

For mastitis milk proteome analysis, the majority of studies have used 2DE followed by MS [7, 8, 47–50]. For example, Hogarth et al. [34] reported down-regulation of caseins, α-lactalbumin and β-lactalbumin while up-regulation of serum albumin and serotransferrin. Similarly, differential expression of proteins including acute phase proteins (APP), lactotransferrin and immunoglobulins was reported during infection [47, 51]. Quantitative analysis of infected milk using isobaric tag for relative and absolute quantification (iTRAQ) has significantly increased protein identifications. For instance, Reinhardt et al. [49] were successful in identifying 2971 proteins significantly expanding the milk proteome. Of these proteins, more than 300 were associated with host defense via neutrophil extracellular traps (NETs) thereby increasing our understanding of mammary gland immune function [52]. Similarly, a number of differentially expressed proteins (e.g. IL-8, IFN-γ) were identified using 2DE in milk collected from post-intramammary infection with *Staphylococcus aureus* [53]. Huang et al. [50] characterized *S. aureus* infected mammary gland using proteomics resulting in the identification of 768 proteins, indicative of the epithelial changes occurring due to infection. Apart from proteins, peptides (n = 154) were identified in mastitis milk caused by *S. aureus* and *Escherichia coli* as potential markers for early and differentially diagnosed mastitis caused by two bacterial sources [8]. Analysis of milk from sub-clinical mastitis revealed changes in abundance of proteins including β-1,4 galactosyltransferase, β-2 microglobulin, complement 3, α-1-acid glycoprotein, and serotransferrin precursor [22]. Inspite of these reported markers, validation of a single biomarker specific to bovine mastitis has not been feasible and presents a unique challenge and opportunity.

The economic consequences of mastitis influences the dairy industry immensely [54]. The cost associated with delayed diagnosis of mastitis includes factors like loss of milk production, discarded milk, veterinary services, labour, product quality, materials and investments, culling and therapeutics [55]. Rollin et al. [56] reported spending of $444 during the first 30 days of lactation, mainly associated with productive losses in milk and culling. Similarly, Cha et al. [57] reported average cost per case associated with different types of mastitis caused by gram-positive, gram-negative bacteria and other clinical mastitic organism to be $133.73, $211.03, $95.31, respectively. Thus, it is critical to adopt a combinatorial approach involving better husbandry and diagnostic methods to monitor animal’s health status including udder before it transitions to clinical mastitis.

Bovine milk has been used for clinical diagnosis, monitoring, control and eradication of infectitious disease such as bovine viral diarrhoea (BVDV) [58]. The causative agent belongs to pestivirus genus and spreads through milk, urine, saliva, nasal discharge, fetal fluids and semen causing acute infection [59]. Infection with BVDV during pregnancy causes huge financial losses as well as increase in incidence of secondary bacterial infections [60]. Furthermore, poor compliance of farmers in implementing control measures has lead to persistence of infection in the herd and spread of virus to uninfected animals within and across herds [58]. Currently, a number of diagnostic tests are available for the detection of virus including enzyme linked immunosorbent assay (ELISA), immunohistochemistry (IHC), reverse transcriptase polymerase chain reaction (RT-PCR), agarose-gel immunodiffusion and viral neutralization test [61]. For example, Gates et al. [58] collected milk samples from female breeding cattle and performed ELISA to detect infected animals. However, there are pitfalls with conventional diagnostic techniques such as higher false positive cases were observed when virus was isolated by culture methods [62]. In addition, due to differences in epidemiology it is pertinent to adopt sensitive detection strategies to identify key molecules that are involved in the pathophysiology of BVDV infection. To this end, MS can play a significant role in qualitative and quantitative characterization of target molecules which could enable clinicians in early disease diagnosis, treatment and control of infection.

During the transition of a pregnant dairy cow from late gestation to early lactation, it experiences a negative energy balance due to rise in demand of milk which cannot be met by feed alone and at risk of developing metabolic disorder known as ketosis [63–65]. This condition is characterized by the increased concentration of ketone bodies such as acetone, acetoacetic acid and β-hydroxybutric acid (βHBA) in blood, milk, urine [66]. Ketosis causes huge financial losses due to treatment cost and decreases in milk production as well as makes the animal susceptible to periparturient diseases such as metritis, mastitis, displaced abomasums [63, 67, 68]. Although a number of diagnostic kits are commercially available, they provide semi-quantitative results [69, 70]. Similarly, the diagnostic test by dipstick using urine have limitations due to difficulty in urine collection compared to milk [71], animal failing to urinate within a reasonable time increasing labour cost [72]. In contrast, accurate measurements from milk by nitroprusside reaction are not sensitive [73]. Currently, the gold standard for diagnosis of ketosis is based on detection of βHBA in serum or plasma using a commercially available instrumentation used in humans for detection of diabetes [74]. However, commercially available kits have not been successful in veterinary practice due to the differences in blood types and antigen expression between humans and animals [75]. Thus, to overcome these limitations, more recently, Weng et al. [75] developed a handheld microfluidic device, which relies on photometric detection of βHBA to confirm ketosis. Similarly, Weng et al. [76] developed quantum dots (QD) to monitor βHBA in cow’s blood and milk. Since newer technologies are being developed for the diagnosis of ketosis with distinct advantages of low cost and detection limits, it is worth trying out the effectiveness of MS-based proteomics to identify as well as validate markers from different biological fluids for routine diagnostic assay in large animal cohort.

Conventionally bovine pregnancy has been detected by palpation per rectum at 60 days after artificial insemination (AI) or ultrasonography at 35 days after AI [77, 78]. However, more recently, 2DE DIGE has been used to separate pregnancy specific proteins from serum [79]. In this study, Lee et al. [79] reported up-regulation of seven protein spots (e.g. modified bovine fibrinogen), while down-regulation of six protein spots (e.g. complement). Similarly, significant change in protein abundance (n = 32) were detected in corpus luteum (CL), an organ formed in the ovary, responsible for the maintainance of pregnancy by 2DE and MALDI [80, 81]. Furthermore, Forde et al. [82] by MS-based proteomics reported 30 unique proteins specific to uterine luminal fluid which could be involved in the interaction between conceptus and the endometrium and potentially play a role in pregnancy detection. Similarly, GC–MS has been extensively used in the detection of volatile compounds from urine of cows and buffaloes. For example, Barman et al. [83] reported identification of six pregnancy-specific compounds such as 2-butenedioic acid-dimethyl, 2-piperidinone, eicosane, nonacosane, octadecanoic acid, butyl ester, and thiazole, 2,4-dimethyl. Thus, unlike traditional approaches, MS can provide an accurate, rapid and non-invasive method of determining pregnancy and can be a valuable tool in improved management of bovine pregnancy.

Quality of milk is a major issue to the dairy industry including consumers due to deliberate addition of adulterants like vegetable fats and oils, melamine and nitrogen-containing compounds like urea and anhydrous milk products such as milk protein concentrate, caseins and whey proteins to milk [84–87]. Furthermore, adulteration of high-value goat and buffalo milk with low priced bovine milk due to easy accessibility has also been found to be an area of foremost concern [88, 89]. These unethical practices have led to serious health concerns to consumers due to addition of unknown allergens. Thus, to check milk adulterants, and safeguard the interests of consumers, different strategies such as polymerase chain reaction (PCR) [89, 90], high performance liquid chromatography (HPLC) [91], infrared spectroscopy [92], immunoassays, nuclear magnetic resonance (NMR) [93] and electrophoretic methods like capillary electrophoresis (CE), urea-polyacrylamide gel electrophoresis [94, 95] have been used. Recently, bovine milk adulteration with goat cheese was successful by amplification of species-specific ribosomal RNA by PCR [89]. Infrared spectroscopy has provided a non-destructive fingerprinting approach to examine and quantify adulterants like whey, urea, caustic soda and hydrogen peroxide in milk [87]. Although these techniques are effective, but have few limitations like co-elution of major proteins in HPLC from bovine, caprine, ovine and buffalo milk leading to inadequate protein identifications [91]. Furthermore, electrophoretic techniques alone cannot differentiate overlapping species specific low abundant proteins [95].

However, recently a number of studies reported the application of MS-based proteomics in detection of adulterations [96, 97]. For example, MALDI-TOF mass spectrometric determination of adulterated milk was found to be a rapid, more competent and cost effective technique. Calvano et al. [88] reported the use of phospholipids as markers of bovine milk adulteration using MALDI-TOF. Similarly 2DE gels coupled with MALDI-TOF has enabled to detect cow milk adulteration in mixtures of buffalo, yak, camel milk mixtures by observing the distribution patterns of α-lactalbumin and β-lactoglobulin, and α_s1_-casein [95]. In addition, adulteration of milk with vegetable fats and oils was identified by MALDI-TOF MS by studying the intact triacylglycerols (TAG) profile [85]. Similarly, LC–MS has been used to estimate the profitable adulteration caused by nitrogen containing compounds like melamine, biuret and urea-based fertilizers in milk allowing detection of contaminants up to 0.5 ppm [86]. More recently, MALDI-TOF was used to analyze antibiotic like benzyl penicillin in dairy milk, using titanium oxide (TiO_2_) nanowires as solid matrix [97]. Thus, the applicability of MS in examining milk quality is increasing due to its rapid and robust screening and characterization of adulterants with minimum sample preparation and does not require any prior modification and derivatization.

### MALDI biotyping

Traditionally, phenotypic properties of microorganisms have been identified by antigen–antibody reaction, Gram staining, and colony morphology, while genotypic traits were characterized using PCR, pulsed-field gel electrophoresis (PFGE), multilocus sequence typing (MLST), restriction fragment length polymorphism (RFLP), and microarrays [98, 99]. Nevertheless, most of these methodologies are expensive, time consuming, laborious, require special skills and are unsuitable for use in routine clinical laboratories. Due to these limitations, MALDI-TOF has recently gained momentum and revolutionalised clinical microbiology laboratories across the world in identifying bacteria, yeast and fungi directly from colonies which were previously misidentified thereby reducing the time for secondary phenotypic identifications [100]. The principle of MALDI-TOF based biotyping relies on unique ribosomal protein profiles matched to a database [101–104]. Genus level identification of unknown microbes is performed by matching peptide mass fingerprint (PMF) with PMFs of known isolated in the database [99]. For species level identification of microorganisms, a spectra of mass range 2–20 kDa represented by abundant ribosomal proteins is matched with PMFs of ribosomal proteins in the database [105]. For example, classical procedures used for detection of *Listeria monocytogenes* take at least 1 week, while MALDI-TOF confirmed by rapid and sensitive analysis within 4–5 h, expanding the applicability of MALDI-TOF for the identification of pathogens [106, 107]. Similarly, detection of antimicrobial resistance using MALDI-TOF has been reported for *S. aureus*, *Acinetobacter baumannii*, and *E. coli* [108, 109]. In addition, MALDI-based identification has been used in biodefense and environmental microbiology and epidemiological studies [110]. In spite of the progress, it must be noted that identification of organisms is database dependent, which are commercially available limiting researchers accessing the ever increasing in size and regularly updated database with discovery of new microbial species. For example, Carbonnelle et al. [111] reported inability of identification of few microorganism due to absence of the organism in the database and not due to methodological error. However, to overcome these limitations, a number of open-source softwares and databases such as mMASS [112], pkDACLASS [113], MALDIquant [114], SpectraBank [115] and BIOSPEAN [116] are freely available.

For human studies, MALDI-TOF based identification of clinical isolates has been extensively used but limited explorations have been performed in veterinary microbial diagnostics. Consequently, the benefit of MALDI-TOF can be used to routinely monitor milk microbiota, create a repository of existing and emerging microorganism, and perform surveillance for dissemination of pathogens in preventing an outbreak including regular screening of milk microbiota for its quality to improve milk as a functional food.

### Lipid markers

To date, the majority of the studies have focused their efforts on analyzing different components of milk such as whey proteins [16–18, 117], milk fat globule membrane (MFGM) [118, 119], and milk exosomes [49, 52], while minimal focus has been on milk lipids. For example, the role of different exogenous and endogenous factors influencing the composition of a particular lipid, a potential source of functional food is limited in both cows and buffaloes. Consequently, researchers and dairy industry are keen to study lipids and its numerous fatty acids (FAs) due to its potential in early disease diagnosis and altering different components to enhance milk quality [120].

Gas chromatography coupled mass spectrometry (GC–MS) has been used to identify milk lipids and FAs as fatty acids methyl esters (FAME) [121]. Stefanov et al. [121] using dichloromethane-ethanol as a solvent identified 49 FAs from bovine milk. Furthermore, Feng et al. [122] identified 108 FAs from milk using a CP-SIL column, while Delmonte et al. [123] were successful in enhancing separation of short-chain FAs and poly-unsaturated fatty acids (PUFAs). In addition, GC has been successfully applied to differentiate FAs based on their positional and geometrical isomers (e.g. isomers of conjugated linoleic acid) [124]. Nevertheless, there are inherent limitations of GC–MS, such as underivatization of FAs into FAME, formation of artifacts and conversion of cis to trans form FAs which in turn alters the composition of fat, during the process of esterification and leads to low lipid recovery and erroneously identified peaks [125].

Recently, MALDI-TOF has been progressively used to study milk lipids as it does not require an additional step of derivatization and results in rapid, accurate detection of lipids. In addition, application of new matrices such as 2,5-dihydroxybenzoic acid, 9-aminoacridine results in well resolved spectra allowing easy characterization of different lipid classes [126]. Similarly, Calvano et al. [127], have characterized phospholipids as species-specific markers in bovine milk using a new matrix, α-cyno-4-chlorocinnamic acid (CCICA). In addition, MALDI-TOF has proven to be a reliable method for high throughput forensic screening of adulterated bovine milk sample with vegetable fats [128]. However, in order to achieve comprehensive lipidome coverage, research groups have used LC–MS/MS [16]. For example, Sommer et al. [129] validated identification and quantification of previously unidentified FAs using LC–MS/MS. Similarly, previously unidentified short chain FAs from cow milk and milk products were identified by LC–MS/MS [130]. Furthermore, significant insights about structural aspects of FAs were reported using LC–MS/MS [131]. In addition, Liu et al. [131] reported a new LC–MS method using a HILIC column for characterization of phospholipids. More recently, MS-based techniques have been used to characterize FAs present in trace amounts in cow milk to maximize the compositional differences between milk samples analyzed across different seasons, lactation periods for identification of potential markers indicative of healthy and pathological condition of secretory cells [132].

### Milk fat globule membrane

Bovine milk fat is dispersed in the form of spherical droplets or globules in the aqueous phase of milk and are found abundantly in milk secreting cells of mammary gland varying in size between 0.2 and 15 µm [133, 134]. The cytoplasmic lipid droplets are made of TAG and encapsulated by membrane of epithelial cell of lactating mammary gland are called as MFGM [135]. The size and distribution of MFGM is influenced by factors such as lactation, age, season, bacteriological quality of milk and breeds [134]. This three-layered complex has been reported to be functionally and nutritionally active as it contains membrane specific proteins including glycoproteins, phospholipids and bioactive sphingolipids [136–138].

The MFGM contains a unique composition of polar lipids and membrane proteins which not only is intriguing as a model to study membrane lipids and proteins but function as markers of biological processes of the cow’s udder cells [139]. For example, Reinhardt et al. [49] reported accumulation of host defense proteins and presence of NETs in MFGM preparations indicative of biology and immune function of the infected mammary gland [49, 140–143]. Similarly, comparative profiling of milk lipids and proteins in healthy versus disease conditions showed contrasting expression of serpin A3-1, vitronectin-like protein and complement factor H [8, 144]. Furthermore, differential expression of lipids and proteins have been used as potential markers (e.g. vitronectin, prostaglandin-D synthase) and presence of oxidative stress response serum amyloid A (SAA) for early detection of mastitis [48, 132, 144, 145]. The presence of phospholipids (e.g. sphingolipids, phosphatidyl ethanolamine) in the MFG membrane imparts a zeta potential to the globules [146, 147], which changes upon contact with reactive oxygen species released by bacteria in infected milk [144]. Thus, MFGM in practice can be used as a valuable tool to test sub-clinical mastitis [49].

### Exosomes

Exosomes are small heterogeneous, extracellular organelles approximately 40–100 nm in diameter [148, 149] found in a variety of body fluids such as blood [150], saliva [151, 152], urine [153], milk [52, 154, 155], and bronchoalveolar lavage fluid [156]. Exosomes contain ubiquitous and cell specific molecules such as proteins, lipids, miRNA and mRNA mediating diverse biological functions, including antigen presentation, signaling, immune function and a source of biomarkers for disease [157–160].

Bovine milk exosomes were partially characterized by Plantz et al. [161], however technical advances in isolation and purification methodology has led to their successful characterization. For example, Reinhardt et al. [49] identified 2350 proteins from exosomes by MS-based proteomics significantly expanding the milk proteome. Of these proteins, a number of proteins were identified as part of neutrophil extracellular trap (NETs) suggesting their role in defense and mammary immune function in mastitis [49]. Exosomes are also reported to be involved in the transmission of pathogens including *Leishmani* spp. and human immunodeficiency virus [162, 163]. Furthermore, different proteins from exosomes such as cytokines, growth factors, hormones, and IgA have been reported to play a significant role in the development of neonatal intestine [164], stimulate secretion of intrinsic growth factors [165] and protection against infection [166]. Taken together, it is also conceivable that exosomal proteins could play an important role to better understand lactation physiology, defense, milk composition and abundance indicative of health and disease.

## Mass spectrometry-based proteomic approach for sample analysis

### Sample preparation and identification

Sample preparation is the most critical and challenging step in proteomics. The sample must be cleaned-up and/or fractionated at the protein or peptide level to unmask medium and low-copy proteins to identify potential markers. Along these lines, different depletion strategies have been used to separate abundant proteins in cow’s milk [21, 118] and urine [10] samples. Subsequently, 2DE has been mainly used to document changing patterns of protein followed by their identification by MS, however, it is limited due to its dynamic range and poor reproducibility [167, 168].

### Qualitative protein identification

Protein identifications can be carried out by tandem mass spectrometry (MS/MS) using TOF/TOF analyzer, with peptide fragmented by post source decay [169] or collision induced dissociation (CID) [170]. However the generation of singly charged peptides by MALDI-TOF leads to preferential cleavage of the peptide backbone with loss of sequence information [171]. This kind of fragmentation may not be a problem for protein identification using adequate software analysis, but can lead to ambiguous protein identification by de novo sequencing. Alternatively, tandem mass spectrometry can be carried out for protein identification using hybrid mass analyzers, such as a combination with quadrupole-time of flight (Q-TOF). In this method, C18 is interfaced as on-line reversed-phase (RP) microcapillary liquid chromatography (LC) electrospray ionization (ESI) [172] or nano-ESI [173] greatly increasing sensitivity, efficiency, and analysis of small sample volume [174]. In this instrument, fragmentation occurs in a predictable manner between the amino acids bonds, enabling identification using software, such as MASCOT [175] or SEQUEST [168]. LC–MS/MS generates multiply charged peptide ions which readily fragment generating high quality and informative tandem mass spectra for confident protein identification [176].

For large scale proteomic analysis, multidimensional protein identification technology (MudPIT) holds great potential. In this technique, a strong cation exchange resin is in line with the RP column. Digested peptides are eluted onto the column at low pH facilitating binding to the cation exchange column and subsequently salt steps are used in an incremental manner to elute peptides onto the C18 RP column for further analysis by MS.

**Fig. 2:**
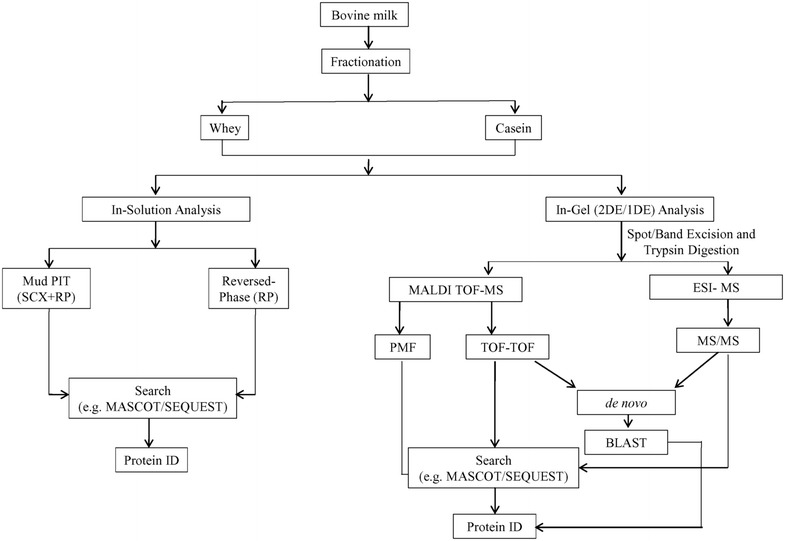
Possible pathways for protein identification. A combination of strategies for characterization of bovine milk whey and casein for maximizing successful protein identification

If the above techniques fail to provide any positive protein identifications, de novo sequencing followed by BLAST searching provides an alternative identification strategy [177, 178]. By this analysis the amino acid sequence is obtained by evaluating the mass difference between two adjacent y- and b-ion series in the fragmentation spectra of the precursor ion [173]. Alternatively, specialized software can be used to create amino acid sequence to interpret tandem mass spectra of peptides [178]. Figure 2 shows possible ways of sample analysis to maximize confident protein identification.

### Quantitation

In addition to protein identifications, MS can be used to quantify differential expressed proteins between two different conditions (healthy vs. disease) either by a label-free or a labeled approach (incorporation of stable isotopes). To date, a number of studies have been performed to quantify milk proteins. For example, mastitis milk proteins were quantified either using a label-free approach [179, 180], or by isobaric tags for relative and absolute quantification (iTRAQ) [49, 50, 118, 142]. In contrast, limited studies have been performed using other labeling techniques such as peptide labeling via metabolic incorporation into cell or tissue culture (15N/14N), stable isotope labeling by amino acids in cell culture (SILAC), amino group labeling using isotope-coded affinity tags (ICAT), tandem mass tags (TMT) and enzymatically catalyzed incorporation (18O labeling).

For targeted quantification of potential markers, although techniques such as ELISA and Western blots are most commonly used, there are limitations including availability, sensitivity and specificity of antibodies for proteins, and multiplexing immunoassays in large animal populations [181]. However, to overcome these limitations, targeted quantification of markers, either by label-free or isotope labeling, can be performed using triple-quadrupole mass spectrometers by single reaction monitoring (SRM) or multiple reaction monitoring (MRM).

## Conclusion

The dairy industry in India has progressed steadily and is the world’s largest milk producer. More recently, there has been an increase in awareness of consumers about milk quality from a health perspective, while little attention has been paid to changing individual constituents due to environmental and physiological factors for enhanced beneficial effect. These compositional variations add to the complexity and diversity of different milk components providing a compelling reason to investigate their changes in abundance for their beneficial effect and markers for early disease diagnosis for timely therapeutic intervention and subsequently diverting attention to better management practices.

From a MS-based proteomic analysis perspective, it is critical and imperative for researchers to combine strategies to increase the likelihood of maximizing positive protein identification. For example, a high-throughput approach for discovery will enable analyses of samples collected from much larger populations followed by a targeted quantification to validate potential markers. Taken together, results of a number of early studies on milk proteomics have reported promising results and also present a challenge to further develop effective proteomic tools for improving livestock productivity and fertility.
